# Anticoagulants beyond coagulation: a narrative review of cardiovascular effects

**DOI:** 10.1093/cvr/cvag043

**Published:** 2026-02-21

**Authors:** Annisa Aarts, Hugo ten Cate, Arina J ten Cate-Hoek

**Affiliations:** Thrombosis Expertise Center, Heart+Vascular Center, Maastricht University Medical Center, P.O. Box 5800, 6202 AZ Maastricht, The Netherlands; Cardiovascular Research Institute Maastricht, Maastricht University, P.O. Box 616, 6200 MD Maastricht, The Netherlands; Thrombosis Expertise Center, Heart+Vascular Center, Maastricht University Medical Center, P.O. Box 5800, 6202 AZ Maastricht, The Netherlands; Cardiovascular Research Institute Maastricht, Maastricht University, P.O. Box 616, 6200 MD Maastricht, The Netherlands; Department of Internal Medicine, Maastricht University Medical Center, P.O. Box 5800, 6202 AZ Maastricht, The Netherlands; Thrombosis Expertise Center, Heart+Vascular Center, Maastricht University Medical Center, P.O. Box 5800, 6202 AZ Maastricht, The Netherlands; Cardiovascular Research Institute Maastricht, Maastricht University, P.O. Box 616, 6200 MD Maastricht, The Netherlands

**Keywords:** Anticoagulants, Heparin, Vitamin K antagonists, Direct oral anticoagulants, Pleiotropy

## Abstract

Anticoagulants are widely used to prevent and treat thrombotic disorders, and continuous advances have refined their efficacy and safety. Beyond their established anticoagulant function, growing evidence indicates that some agents exert additional cardiovascular effects through pleiotropic mechanisms. In this narrative review, we examine both the intended and potential unintended effects of anticoagulants, with particular attention to their actions beyond coagulation. We discuss their influence on vascular pathology and cardiac function, highlighting distinctions between different anticoagulant classes. A deeper understanding of these pleiotropic effects may help guide future research, improve long-term outcomes, and support more targeted therapeutic strategies for patients most likely to benefit.

## 1. Introduction

Anticoagulants inhibit the coagulation cascade to prevent or treat thrombosis. Historically, two main classes emerged in the early 20th century. The first comprised unfractionated heparin (UFH), glycosaminoglycans of variable chain length purified from animal tissue, which accelerate antithrombin (AT)-dependent inactivation of serine proteases, including FXa and thrombin. The next generation included low molecular weight heparins (LMWHs), less heterogeneous and enriched in AT-binding capacity, and the synthetic pentasaccharide fondaparinux.^1^

The second class of oral anticoagulants (OACs) consisted of vitamin K antagonists (VKAs), based on the discovery that coumarins—derived from dicoumarol in spoiled sweet clover—inhibit vitamin K-dependent γ-carboxylation of coagulation proteins.^2^ Over half a century later, direct OACs (DOACs) were developed as small molecules targeting FXa or thrombin. More recently, FXIa inhibitors have emerged as a novel class with potential safety advantages. In addition, RNA silencing therapies and monoclonal antibodies, mostly targeting FXI(a), are in (pre)clinical testing.^3^

While anticoagulants aim to prevent thrombosis, their principal unintended effect is bleeding. Both efficacy (thrombus inhibition) and safety (bleeding risk) depend on anticoagulation intensity. Beyond fibrin inhibition, anticoagulants can also modulate fibrinolysis, platelets, and endothelial function, contributing to both antithrombotic and bleeding-prone actions, as well as pleiotropic effects, including anti-inflammatory properties. This review examines the intended and unintended cardiovascular effects of current anticoagulants.

## 2. Pharmacological properties of anticoagulants

### 2.1 Parenteral anticoagulants: LMWHs and direct thrombin inhibitors

UFH and LMWH act by amplifying the rate of coagulation protease inactivation by the natural serine protease inhibitor AT.^4^ Binding of UFH to AT results in a conformational change in the latter, thereby rendering it a rapid and irreversible inhibitor of FXa and thrombin, and probably also of FIXa, FXIa, and FXIIa. Subsequently, heparin dissociates from the complex for reuse.^1^ This mechanism is responsible for the largest anticoagulant effect. The molecular weight of heparin is variable, and only one-third of UFH molecules possess the unique AT-binding site.

Two additional mechanisms of action have been described. Firstly, at higher concentrations (≥5 units/mL) and in the presence of heparin cofactor II, heparin can catalyse thrombin inhibition without the AT-binding site.^5^ Secondly, at very high concentrations, heparin inhibits FXa activation by binding FIXa, independently of AT and heparin cofactor II.^6^

Heparin is administered parenterally as it is not absorbed from the gastrointestinal (GI) tract.^1^ Bioavailability with subcutaneous administration is lower compared with intravenous administration (*Table 1*).^4^ Owing to its strong negative charge, UFH molecules associate with the vascular endothelium, releasing proteins, such as lipoprotein lipase that are physiologically attached to endothelial glycosaminoglycans, including heparan sulfate.^14^

**Table 1:** Pharmacological properties of parenteral anticoagulants

	UFH^6,7^	LMWH^6,8^	Fondaparinux^4,9^	DTI^6,10^ Argatroban (A) Bivalirudin (B)	FXI inhibitor^3^ Antisense oligonucleotides (A) Monoclonal antibodies (M)
Target	Antithrombin	Antithrombin	Antithrombin	FIIa	FXI(a)
Prodrug	No	No	No	No	No
Route	Intravenous Subcutaneous	Subcutaneous	Subcutaneous	Intravenous	Intravenous (M) Subcutaneous (AM)
Daily doses	Variable, continuous Fixed, twice	Fixed, once/twice	Fixed, once	Variable, continuous	Fixed, weekly-monthly
Monitoring	aPTT Anti-FXa	Anti-FXa	Anti-FXa	aPTT	aPTT
Antidote	Protamine	Protamine (partial)	^a^	^a^	^a^
Target effects	Inhibition of FIIa and FXa	Inhibition of FXa and limited inhibition of FIIa	Inhibition of FXa	Inhibition of FIIa	Inhibition of intrinsic coagulation pathway
Unintended effects (other than cardiovascular)	Osteoporosis due to binding osteoblasts and osteoclasts Anti-inflammatory properties because of ligand interactions^11^	Lower osteoporosis risk compared with UFH Anti-inflammatory properties because of ligand interactions^11^	–	Downregulation of inflammatory cytokines (A)^12^	–
Bioavailability (%)	100 (intravenous) 10–40 (subcutaneous)^13^	90	100	100	100 (intravenous)
Peak serum concentration (h)	Immediate (intravenous)	3–5	2	–	0.1–6 (AM) 7–21 days (M subcutaneous)
Plasma protein binding (%)	High, variable	Less than UFH	97–98.6	54 (A) 0 (B)^13^	–
Volume of distribution	0.07 L/kg	Close to plasma volume	7–11 L	0.2 L/kg (A)	–
Half-life (h)	0.5–2.5 (intravenous)	3–6	17	0.5–1	11–121 (M) 2–6 weeks (AM)
Renal excretion (%)	Variable	Predominant	64–77	0 (A) 20 (B)	Minimal (M)

aPTT, activated partial thromboplastin time; DTI, direct thrombin inhibitor; F, factor; h, hour; kg, kilogram; L, litre; LMWH, low-molecular-weight heparin; UFH, unfractionated heparin; –, not reported.

^a^No specific antidote available.

Heparin clearance is biphasic: rapid cellular uptake by endothelial cells and macrophages, followed by slower renal elimination. Clearance is dose-dependent, leading to a variable half-life.^15^ In addition, the anticoagulant response varies between patients. Therefore, the anticoagulant effect should be monitored using the activated partial thromboplastin time (aPTT) or an anti-Xa assay. In cases where heparin doses are higher (e.g. in cardiopulmonary bypass surgeries), the activated clotting time is used. The anticoagulant effect of heparin is rapidly reversed by administration of protamine.^1^

Depolymerizing UFH to about one-third of its molecular weight produces LMWH. Smaller LMWH fragments (<18 saccharide units) cannot bind both AT and thrombin simultaneously; thus, LMWH primarily targets FXa.^1^

Compared with UFH, LMWH binds less to plasma proteins and cells, resulting in more predictable pharmacology, a longer half-life, and stable anticoagulant effects (*Table 1*). Its predictable dose-response allows weight-adjusted dosing in most patients,^6^ and reduced endothelial binding enhances subcutaneous bioavailability.^8^

LMWHs are mainly cleared renally; impaired renal function prolongs half-life, sometimes requiring dose adjustment based on peak anti-Xa levels. Their anticoagulant effect is only partially reversed by protamine.^6^

UFH is primarily used in emergent vascular interventions or cardiopulmonary bypass, while LMWH is common for prophylaxis and treatment of venous thromboembolism (VTE), and in patients for whom OACs are contraindicated, such as during pregnancy or in cancer (*Table 2*).

**Table 2:** Recommended anticoagulant(s) per indication

Indication	Recommended anticoagulant(s)	Rationale
Antiphospholipid syndrome	VKA	Best balance between efficacy and risks^16^ LMWH in case of contraindication for VKA, but long-term use might be inconvenient^16^ Increased risk of arterial thrombosis with DOAC compared with VKA^16^
Atrial fibrillation	DOAC or VKA	Similar efficacy with lower risk of (notably intracranial) bleeding with DOAC compared with VKA^17^ VKA are indicated in moderate-severe mitral stenosis, where DOAC exhibit inferior outcomes^17^ VKA in case of contra-indication for DOAC^17^
Cancer-associated thrombosis	Direct factor Xa inhibitor or LMWH	Reduced recurrence rate but increased nonmajor bleeding risk with direct factor Xa inhibitor compared with LMWH, especially in gastrointestinal and genitourinary malignancies^18^ Improved efficacy over VKA^18^
Heparin-induced thrombocytopenia	Argatroban, bivalirudin, danaparoid, fondaparinux, or DOAC	Choice of non-heparin anticoagulant depends on drug factors (e.g. availability), patient factors, and experience^19^ Argatroban or bivalirudin may be preferred in critically ill patients because of their short half-life^19^ Fondaparinux, danaparoid, and DOAC (rivaroxaban most studied) may be more convenient to administer in clinically stable patients^19^ VKA increase the risk of limb gangrene by reducing protein C levels^20^
Left ventricular thrombus	VKA, LMWH, or DOAC	DOAC seems to be a reasonable alternative to VKA, especially if managing VKA therapy is difficult^21^
Mechanical heart valves	VKA	Reduced thrombus risk due to prosthetic material and abnormal flow conditions^22^ DOAC not approved^22^
Portal vein thrombosis in liver cirrhosis	VKA, LMWH, or DOAC	VKA, LMWH, and DOAC can be used in CTP Class A and B cirrhosis, only LMWH can be used in CTP Class C cirrhosis^23^ INR monitoring for VKA is unreliable^23^
Post-operative thromboprophylaxis	LMWH, UFH, or DOAC	LMWH or UFH preferred after major general surgery^24^ DOAC preferred after total hip or knee arthroplasty, otherwise LMWH^24^
Anticoagulation during pregnancy	LMWH	Lowest foetal risks^25^ Risk of embryopathy with VKA, mainly in first trimester^25^ DOAC contra-indicated, no adequate studies available^25^
Venous thromboembolism	DOAC	Similar efficacy with lower risk of (notably intracranial) bleeding with DOAC compared with VKA^26^

CTP, Child-Turcotte-Pugh; DOAC, direct oral anticoagulant; INR, international normalized ratio; LMWH, low-molecular-weight heparin; UFH, unfractionated heparin; VKA, vitamin K antagonist.

Fondaparinux is a synthetic pentasaccharide containing only the AT-binding site, selectively enhancing FXa inhibition. With a half-life of ∼17 h, once-daily subcutaneous dosing achieves therapeutic levels. Its clearance is almost entirely renal, so it is contraindicated in severe renal insufficiency. Monitoring can be done with anti-FXa assays. Although no specific antidote exists, recombinant FVIIa may partially reverse its effect.^4^

Unlike UFH, LMWH, and fondaparinux, direct thrombin inhibitors (DTIs) inhibit thrombin independently of AT. Approved parenteral DTIs include bivalirudin and argatroban. Hirudin, the natural prototype, inspired the shorter synthetic analogue bivalirudin.^4^ Bivalent DTIs (hirudin, bivalirudin) bind thrombin at both its active site and exosite 1, while univalent DTIs (argatroban) bind only the active site. DTIs inhibit both soluble and fibrin-bound thrombin.^27^

Bivalirudin is metabolized by proteolysis and hepatic metabolism, though renal impairment may prolong its half-life. Argatroban is primarily metabolized by the liver, requiring dose adjustment in hepatic dysfunction.^4^ The anticoagulant effect of DTIs is monitored by aPTT, particularly in patients at high bleeding risk, as no specific antidote is available.^27^

### 2.2 Vitamin K antagonists

Vitamin K is a coenzyme essential for synthesizing vitamin K-dependent coagulation proteins (FII, FVII, FIX, FX, protein C, S, and Z) and several other hepatic and extrahepatic proteins. It undergoes continuous recycling via reduction and oxidation, mediated by the vitamin K epoxide reductase complex subunit 1 (VKORC1). VKAs inhibit VKORC1, inducing functional vitamin K deficiency in hepatocytes and depleting active coagulation factors.^28^ Consequently, their anticoagulant effect can be reversed by administering vitamin K. VKAs also affect extrahepatic vitamin K-dependent proteins—17 are known—including osteocalcin, matrix Gla protein (MGP), and growth arrest-specific 6 (Gas6).^29^

VKAs are administered orally. Due to the long half-lives of FII and FX, several days of concurrent heparin are required to reach the narrow therapeutic window.^28^ Management of VKAs is challenging due to individual variability and interactions with medications and diet, requiring dose adjustments based on the international normalized ratio (INR), a standardized measure of prothrombin time (PT).^30^ Fiix-assay studies—suggesting that FII and FX predominantly determine thrombin generation under VKA treatment and that FVII exhibits marked variability—indicate that measuring FII and FX instead of the INR may provide more stable anticoagulation, but this assay is not yet routinely applied.^31,32^

VKAs are rapidly absorbed in the stomach and small intestine, with almost complete oral bioavailability. Plasma protein binding is high, resulting in a low free plasma concentration that is pharmacologically active.^30,33^ Acenocoumarol has the shortest half-life, followed by warfarin and phenprocoumon (*Table 3*). The long half-life of phenprocoumon is attributed to slower CYP-mediated clearance, as well as enterohepatic recycling.^33^

**Table 3:** Pharmacological properties of oral anticoagulants

	VKA^30,33^ Acenocoumarol (A) Phenprocoumon (P) Warfarin (W)	DOAC^34–37^ Apixaban (A) Dabigatran (D) Edoxaban (E) Rivaroxaban (R)	FXI inhibitor^38,39^ Small molecules Asundexian (A) Milvexian (M)
Target	Vitamin K epoxide reductase	FXa (AER) FIIa (D)	FXIa
Prodrug	No	No (AER) Yes (D)	No
Route	Oral	Oral	Oral
Daily doses	Variable, once	Fixed, once (ER) Fixed, twice (AD)	Fixed, once/twice
Monitoring	INR	Anti-FXa (AER) Plasma diluted thrombin time (D)	–
Antidote	Vitamin K PCC	Andexanet alfa (AR) Idarucizumab (D)	–
Target effects	Depletion of vitamin K-dependent clotting factors	Inhibition of thrombin generation (AER) Inhibition of fibrin clot formation (D)	Inhibition of intrinsic coagulation pathway
Unintended effects (other than cardiovascular)	Cartilage calcification due to inactivation of matrix Gla protein^40^ Accelerated bone loss and osteoporosis due to dysfunction of osteocalcin^2^	Modulation of inflammation via PAR-signalling^41^	–
Bioavailability (%)	60 (A) 90 (P) 100 (W)	50 (A) 6.5 (D) 62 (E) 80–100 (R)	100 (A)
Peak serum concentration (h)	1–3 (A)^13^ 48–72 (P)^13^ 0.3–4 (W)	3–4 (A) 0.5–2 (D) 1–2 (E) 2–4 (R)	1–4
Plasma protein binding (%)	>98 (A)>99 (PW)	87 (A) 35 (D) 55 (E) 92–95 (R)	–
Volume of distribution	0.22–0.52 L/kg (A) 0.11–0.14 L/kg (P) 0.11–0.18 L/kg (W)	21 L (A) 60–70 L (D) 107 L (E) 50 L (R)	70.5 L (A) 347 L (M)
Half-life (h)	6.6 (A) 110–125 (P) 35–58 (W)	12 (A) 11 (D) 10–14 (E) 5–13 (R)	14–18 (A) 8–18 (M)
Renal excretion (%)	65 (A) 65 (P) 80 (W)	27 (A) 85 (D) 35 (E) 33 (R)	20 (A) 7–18 (M)

DOAC, direct oral anticoagulant; F, factor; h, hour; INR, international normalized ratio; kg, kilogram; L, litre; PAR, protease-activated receptor; PCC, prothrombin complex concentrate; VKA, vitamin K antagonist; –, not reported or unknown.

Drugs metabolized via CYP2C9 and CYP3A4 may alter VKA plasma concentrations, increasing bleeding risk, or reducing efficacy.^42^ As VKAs are primarily cleared hepatically, they are considered safe in end-stage renal disease.^30^ In emergencies, anticoagulation can be rapidly reversed with, preferably four-factor, prothrombin complex concentrate (PCC).^42^

### 2.3 Direct OACs

DOACs reversibly bind the active site of FXa (apixaban, edoxaban, rivaroxaban) or FIIa (dabigatran), thereby inhibiting thrombin generation and fibrin formation. Peak plasma concentrations occur within hours of oral intake (*Table 3*).^34–37^

Dabigatran is a prodrug with low bioavailability due to lipophilicity and pH-dependent absorption; tartaric acid in the formulation enhances uptake.^43^ Other DOACs have higher bioavailability due to better solubility and less pH dependence. Half-lives are relatively short, up to 14 h. Renal excretion varies from 27% for apixaban to 85% for dabigatran.^34–37^

After absorption, DOACs enter the mesenteric circulation or are pumped back into the intestinal lumen by P-glycoprotein (P-gp) transporters.^44^ These efflux pumps, expressed in tissues with excretory or barrier functions, limit absorption, enhance elimination, and prevent distribution to sensitive organs like the brain.^45^ Cytochrome P450 enzymes also play an important role in the clearance of apixaban and rivaroxaban.^46^ Consequently, co-administration with strong P-gp or CYP3A4 inducers or inhibitors can lead to subclinical concentrations or increased bioavailability and subsequent thrombosis or bleeding risk, respectively, and is therefore contraindicated.^46^ Apixaban and rivaroxaban are also substrates of the breast cancer resistance protein, which functions similarly to P-gp.^47,48^

Routine laboratory monitoring and dose adjustment are not necessary given their stable pharmacokinetics. However, DOAC concentrations can be measured directly via mass spectrometry or indirectly using anti-FXa assays (for FXa inhibitors) or diluted thrombin time (for dabigatran).^49,50^ Several point-of-care assays are currently under investigation for emergency use.^51^

Despite fixed dosing, DOAC plasma concentrations can vary substantially between individuals, potentially increasing thrombosis or bleeding risk when plasma levels fall outside the ‘on-therapy’ ranges as established in major clinical trials.^52^ Monitoring may be useful in selected patients—frail elderly, those with impaired GI absorption, extreme weight, or potential drug interactions—where concentrations may deviate from expected ranges.^42,53^

Reversal agents include andexanet alfa (for apixaban, rivaroxaban, off-label edoxaban) and idarucizumab (for dabigatran). Andexanet alfa is a recombinant, inactive FXa; idarucizumab is a monoclonal antibody binding dabigatran with high affinity.^54^ PCCs are often used instead due to cost and safety concerns, as andexanet alfa has been linked to increased thrombotic events in bleeding patients with underlying thromboembolic risk.^55,56^ Only dabigatran can be removed by haemodialysis due to its low plasma protein binding (35%).^46^

OACs are mostly prescribed for the treatment and prevention of thrombosis. Large clinical trials have established DOACs’ comparable or superior efficacy and lower bleeding risk compared with the previous gold standard, VKAs, for the major indications atrial fibrillation (AF) and VTE.^57^ However, VKAs remain preferred in high thrombogenic conditions like mechanical heart valves, AF associated with mitral valve disease, antiphospholipid syndrome (APS, especially with triple-positive antibodies), and recurrent thrombosis while on DOACs (*Table 2*). In frail elderly, there is uncertainty regarding the optimal anticoagulant for stroke prevention in AF; for patients who are stable on VKAs, it may be prudent not to switch to a DOAC.^58,59^

### 2.4 Factor XI-inhibitors

Inhibiting FXI(a) can reduce thrombus formation via the contact activation pathway without inhibiting physiological haemostasis mediated via the tissue factor (TF) pathway. Both oral and parenteral compounds are undergoing clinical testing.^60^

FXI inhibitors fall into three main classes: small molecules, antisense oligonucleotides, and monoclonal antibodies. Others include natural peptides and aptamers. Small molecules are orally administered and reversibly bind to FXIa. Due to their short half-life, daily dosing is required to maintain a therapeutic effect.^3^

Antisense oligonucleotides are administered subcutaneously and act in the liver by degrading FXI mRNA, thereby inhibiting protein synthesis.^61^ The anticoagulant effect develops over several weeks, but their long half-life allows weekly or monthly dosing, improving adherence yet complicating rapid reversal. In emergencies, replacement with fresh frozen plasma or FXI concentrate may be considered.^3^ Their strong plasma protein binding limits renal clearance.^61^

Monoclonal antibodies, administered intravenously or subcutaneously, selectively bind FXI(a) with rapid onset and prolonged half-life, enabling extended dosing intervals. They are metabolized and cleared mainly by phagocytic cells and the reticuloendothelial system. For urgent reversal, recombinant FVIIa may be used; however, available data suggest an increased risk of thromboembolic events in off-label settings—particularly arterial thromboses—though most trials have been underpowered to reliably quantify this risk.^62^

## 3. Intended effects

### 3.1 Anticoagulant and antithrombotic mode of action

**Figure 1:**
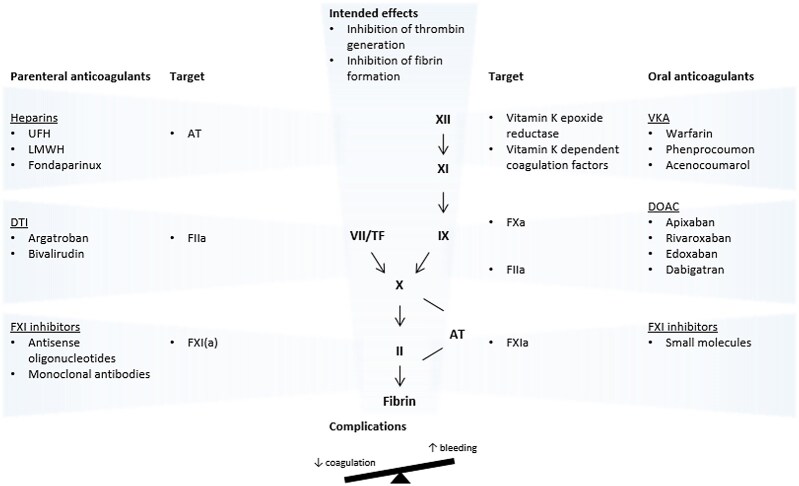
Intended effects of anticoagulants. AT, antithrombin; DOAC, direct oral anticoagulant; DTI, direct thrombin inhibitor; LMWH, low-molecular-weight heparin; TF, tissue factor; UFH, unfractionated heparin; VKA, vitamin K antagonist.

All anticoagulants are administered to prevent fibrin formation and, consequently, limit thrombosis, allowing natural fibrinolytic mechanisms to restore reperfusion of the occluded blood vessel(s) (*Figure 1*). Different anticoagulants achieve inhibition of fibrin formation by different routes, with the common denominator being the inhibition of thrombin formation. Traditionally, clotting assays have been used to study the impact of anticoagulants in plasma, with the aPTT and PT serving as the most sensitive tests for UFH and VKAs, respectively. LMWHs, DTIs, and DOACs have variable effects on clotting assays.

Thrombin generation analysis in plasma has been helpful in clarifying mechanistic differences among anticoagulants. All FXa inhibitors and dabigatran prolong the lag time and time to peak in thrombin generation assays, but only the FXa inhibitors lower the thrombin peak level in a nonlinear, dose-dependent manner.^63^ Within the spectrum of anticoagulants, VKAs appear to have the strongest potential to inhibit thrombin generation in a dose-dependent way, probably because VKAs affect both the extrinsic and intrinsic routes of coagulation. Clinical studies confirm this overall stronger suppression of thrombin generation, based on lower levels of the prothrombin fragment F1 + 2 in patients on VKAs vs. DOACs.^64,65^

Additionally, UFH and, to some extent, LMWH are generally more potent in inhibiting thrombin generation than selective anticoagulants, including DOACs (Shaw *et al*., manuscript under revision). This may be explained by the fact that UFH, like VKAs, inhibits the coagulation cascade at multiple levels. These differences in modes of action may also underlie the superior efficacy of VKAs and heparins in highly thrombogenic settings like APS, mechanical heart valves, and extracorporeal circuits.

### 3.2 Effects on fibrin clot formation and fibrinolysis

Data on the effects of heparins on fibrin clot properties and fibrinolysis remain relatively limited. In patients with acute pulmonary embolism treated with enoxaparin, higher plasma anti-FXa activity was associated with increased clot permeability and shortened clot lysis time.^66^ Another study demonstrated that heparin and LMWH, but not fondaparinux, modified fibrin clot structure, potentially enhancing fibrinolysis.^67^ In contrast to other anticoagulants, fondaparinux increased clot permeability, without a dose-dependent effect.^68^ The absent or modest effect of fondaparinux may be attributed to its selective inhibition of FXa. This hypothesis was tested in an *in vitro* study, which demonstrated that profibrinolytic activity was more closely associated with thrombin inhibition than with anti-FXa activity.^69^ Thrombin exerts antifibrinolytic effects through activation of thrombin activatable fibrinolysis inhibitor (TAFI); inhibition of thrombin activity renders fibrin clot structure more sensitive to fibrinolysis.^69^

Given that fibrin clot formation is dependent on thrombin availability, inhibition of thrombin generation may result in alterations of fibrin clot properties.^70^ Fibrin clots generated in the presence of rivaroxaban and dabigatran were characterized by thicker fibrin fibres and increased permeability compared with clots generated in their absence. In addition, reduced activation of TAFI also contributed to the formation of more lysable clots.^71,72^ Furthermore, rivaroxaban and apixaban have been shown *in vitro* to shorten clot lysis time, possibly by enhancing t-PA cofactor activity and promoting plasmin generation.^73^

## 4. Unintended effects

### 4.1 Impairment of haemostasis and bleeding risk

Clinically, bleeding remains the major and most feared adverse effect of anticoagulant therapy. To date, no anticoagulant completely lacks bleeding-enhancing potential, although inhibition of FXI(a) is anticipated to offer a safer profile. The mechanisms underlying anticoagulant-associated bleeding are still incompletely understood. Established patient-related risk factors include advanced age, renal impairment, concomitant medications, a history of bleeding or stroke, and hypertension; these have been incorporated into various bleeding risk scores.^74^

The quality of anticoagulant management also plays a critical role. For VKAs, the time in therapeutic range remains a key determinant of both efficacy and bleeding risk.^74^ In DOAC users, variability in peak plasma levels is associated with bleeding, while trough levels correlate with thrombotic risk.^42,52^ For all anticoagulants, there is a dose-dependent increase in bleeding risk, highlighting the need to identify the optimal intensity for each patient and indication.

Certain types of bleeding are more often associated with specific classes of anticoagulants. For example, DOACs are associated with a smaller risk of intracranial haemorrhage compared with VKAs, which is possibly related to the multiple target effect of VKAs including on FVII inhibition in the TF-rich brain.^75,76^ Conversely, GI bleeding occurs relatively more often with DOACs (dabigatran, edoxaban, and rivaroxaban) than with VKAs or LMWHs, likely due to their variable concentration of active drug in the GI tract and higher local tissue penetration.^75^ Two out of three women initiating anticoagulation for VTE experience abnormal menstrual bleeding.^77^ Apixaban, dabigatran, and VKAs appear to carry a lower risk of heavy menstrual bleeding compared with rivaroxaban and edoxaban.^78^ However, prospective randomized studies directly comparing different anticoagulants are still lacking.

One underexplored bleeding mechanism relates to endothelial barrier integrity, which may be critically important in situations where this barrier is perturbed, such as in ischaemia-reperfusion injury following ischaemic stroke.^79^ Similarly, in GI or other organ bleeding, local endothelial damage may be a very important contributor or cause of bleeding during anticoagulation. As endothelium integrity is affected by inflammation, oxidation, and trauma, related biomarkers (e.g. growth differentiation factor-15, matrix metalloproteinase-9, vascular adhesion protein-1, serum protein S100B, fibronectin, and tight junction proteins like occludin) may help to better estimate bleeding risk.^79^ Furthermore, biomarkers related to inflammation, vascular remodelling, endothelial cell damage, coagulation, and fibrinolysis (specifically growth differentiation factor-15, cTnT-hs, and seven novel biomarkers) have been independently associated with major bleeding in patients with AF receiving OAC.^80^

### 4.2 Interactions with blood platelets

Given the inherent risk of bleeding associated with anticoagulation, numerous studies have explored potential interactions with platelet count and function. Thrombocytopenia, as frequently observed in patients receiving cancer treatment, increases bleeding risk and necessitates adjustment of anticoagulant management.^81^ Although anticoagulant associated thrombocytopenia is uncommon, the rare occurrence of heparin-induced thrombocytopenia (HIT) remains a serious and feared complication.^20^ The risk of HIT is lower with LMWH and absent with fondaparinux compared with UFH, reflecting reduced binding of shorter heparin chains to platelet factor 4.^6^ Given the diagnostic and therapeutic complexity of HIT, detailed discussion is provided in dedicated expert reviews.^82,83^

Apart from HIT, UFH may, possibly through electrostatic interactions with surface proteins, enhance platelet aggregation, depending on heparin chain length and its affinity for AT.^84,85^ However, these effects are generally of limited clinical relevance, except in the context of extracorporeal circulation, where UFH exposure may contribute to platelet count reduction and functional impairment as part of the broader coagulopathy associated with such procedures.^86^

All anticoagulants affect thrombin-induced platelet activation, one of the most relevant haemostatic pathways *in vivo*. Thrombin is a principal agonist of platelet activation via binding to and activation of protease-activated receptor (PAR) 1 and PAR-4, with different kinetics.^87,88^

Several studies have examined platelet activation pathways in patients receiving warfarin. Platelet aggregation in response to collagen or epinephrine appears unaffected by warfarin therapy and may even be enhanced at higher INR levels.^89^  ^,90^ Increased platelet reactivity has also been reported in patients treated with warfarin for AF or secondary stroke prevention.^91^ In healthy volunteers exposed to subtherapeutic warfarin levels (INR ≈1.5) for 35 days, heightened platelet responses to adrenaline and adenosine diphosphate (ADP), and to a lesser extent to collagen, were observed.^92^ Whether this enhanced platelet responsiveness represents a compensatory mechanism in the context of suppressed thrombin-mediated platelet activation remains uncertain.

In platelet-rich plasma, both FXa inhibitors and DTIs inhibit TF-triggered platelet aggregation in a concentration-dependent manner.^93^ The effect of rivaroxaban on platelet function has been the focus of several studies.^94^  *In vitro*, rivaroxaban inhibited P-selectin expression at the platelet surface upon stimulation of aggregation by thrombin (or TF).^95^ Additionally, FXa inhibition reduced soluble glycoprotein VI (GPVI) concentrations, thereby limiting platelet activation during atherosclerotic plaque rupture, and diminished thromboxane synthesis and Nox-2-dependent oxidative stress following GPVI stimulation.^96^

*In vivo*, rivaroxaban (and apixaban) did not alter platelet responses to a range of agonists in patients with AF.^97,98^ Several clinical studies have shown reduced levels of soluble platelet activation biomarkers, including P-selectin and β-thromboglobulin, in patients treated with rivaroxaban;^99–101^ however, other studies did not confirm this effect.^98^ Rivaroxaban reduced platelet activation, aggregation, and thrombus formation through inhibition of FXa-mediated PAR-1 activation under flow conditions and *in vivo*. This resulted in the formation of less stable thrombi and was associated with increased thrombus fragment embolization in murine models of arterial thrombosis.^102^ Finally, there is also evidence suggesting that rivaroxaban inhibits thromboxane production *in vivo*.^103^

Long-term FXa-inhibition by DOACs has been associated with reduced platelet-mediated thromboinflammation and, consequently, smaller infarct sizes after acute myocardial infarction and stroke. Long-term treatment induces alterations in platelet RNA and proteomic profiles that attenuate platelet granule secretion and thereby reduce neutrophil extracellular trap formation. This effect was verified by single-gene analysis demonstrating downregulation of vesicle-associated membrane protein 8.^104^

The focus on platelet reactivity increased after the RE-LY study observed higher myocardial infarction rates in patients with AF taking dabigatran compared with warfarin.^105,106^ Several studies addressed effects of dabigatran on platelets either in blood from healthy subjects with neutral effects, or in patients with stroke on dabigatran who had higher platelet reactivity to ADP when compared with patients on FXa inhibitors.^107,108^ A study in hospitalized patients showed increased platelet reactivity after starting dabigatran, associated with increased PAR density at the platelet surface.^109^  *In vitro*, addition of a DTI to whole blood reduced the amount of thrombin-induced platelet binding to monocytes or granulocytes, expressing a reduced amount of TF mRNA.^110^ Although dabigatran did not influence PAR-1 expression on platelets from AF patients, expression of the platelet activation marker CD62P was inhibited after thrombin stimulation.^111^ At the same time, chronic dabigatran therapy has been associated with increased thrombin receptor-activating peptide-induced platelet aggregation, as well as enhanced platelet adhesion and thrombus formation due to altered GPIbα interactions.^96^ Overall, these, sometimes conflicting, data do not suggest any consistent effects of dabigatran on platelet reactivity, suggesting that other factors may explain the unexpected higher risk of myocardial infarction. One speculated mechanism is that DTIs impair thrombin-induced protein C activation, causing a temporary hypercoagulability, similar to what may rarely occur with VKAs, but the clinical relevance in this context is unknown.^112^

Given the beneficial effects on clinical outcomes of dual pathway inhibition (DPI) with low-dose aspirin and low-dose rivaroxaban (2.5 mg bd) in the COMPASS and VOYAGER trials,^113,114^ mechanistic studies addressed the addition of low-dose rivaroxaban to antiplatelet therapy. These studies reported reduced thrombus formation without effects on pure platelet-dependent clotting or platelet aggregation measured by light transmission aggregometry. However, the addition of rivaroxaban reduced platelet expression of high mobility group box 1, without a concurrent effect on P-selectin.^115^

In conclusion, most, if not all, OACs appear to inhibit thrombin-induced platelet activation and aggregation, whereas platelet activation by other agonists such as ADP or collagen remains unchanged or may even be enhanced. This phenomenon could represent a compensatory response to reduced thrombin-mediated signalling. Subtle additional platelet-inhibitory effects of certain DOACs, such as rivaroxaban, may contribute not only to their antithrombotic efficacy but also to the increased bleeding risk observed with DPI.

### 4.3 Impact on extracellular vesicles and immune modulation

Extracellular vesicles (EVs) are circulating membrane-derived particles with a messenger function, released by cells in response to activation or apoptosis. In patients with stable cardiovascular disease, DPI was associated with a reduction in EVs from activated platelets, less so from neutrophils, and not from endothelial origin.^116^ Characterization of EV contents showed a subset of differentially expressed proteins, including several of platelet and neutrophil origin, and an overall reduction in inflammasome activation compared with aspirin alone.^116^

In patients with AF, treatment with rivaroxaban compared with warfarin was associated with a less inflammatory proteomic profile in EV-enriched plasma fractions, along with less endothelial activation markers.^117^ Similarly, EVs from patients with VTE managed with either warfarin or rivaroxaban showed a differential expression of six proteins (PROZ, F2, SERPINA10, APCS, F10, and PROS1), with an anticoagulant and anti-inflammatory profile in the DOAC treated subjects.^118^

Potentially, a reduction in EV inflammatory proteome contents, may reflect or have impact on systemic inflammatory activity. In a prospective observational study of patients with stable atherosclerotic disease, DPI was associated with reductions in plasma levels of interleukin-6 (IL-6) and fibrinogen after 24 weeks, compared with baseline.^119^ However, in patients with diabetic atherosclerotic disease, addition of low dose rivaroxaban did not have any detectable effect on endothelial and inflammatory biomarkers.^120^ Moreover, three months DPI treatment in other patients with stable atherosclerotic disease did not reveal any relevant effects on circulating inflammatory mediators or mononuclear cell responsiveness.^121^

In a study comparing AF patients using therapeutic dosed rivaroxaban with warfarin, a trend towards a reduced level of pro-inflammatory cytokines and an increase in chemokines in rivaroxaban treated subjects was seen, with uncertain net anti-inflammatory effect.^122^ In a substudy of the X-VeRT trial on cardioversion in AF patients, biomarkers of coagulation and inflammation were similarly lowered under treatment with both rivaroxaban and VKAs, though F1 + 2 levels remained more elevated under rivaroxaban.^65^ Finally, using a panel of inflammatory markers, 187 patients with AF randomly assigned to rivaroxaban or dabigatran did not yield any differences between these drugs at 12 months follow up.^123^ Summarizing, subtle indications of anti-inflammatory effects of DOACs (mostly rivaroxaban) were found in circulating EVs without evident changes in systemic inflammatory biomarkers; a similar effect, if any, of VKAs or dabigatran cannot be excluded.

### 4.4 Endothelium

Vascular endothelial cells play a central role in regulating coagulation by maintaining a balance between procoagulant and anticoagulant mechanisms.^124^ This regulation occurs in part through the uptake and metabolism of endogenous glycosaminoglycans, as well as the uptake of exogenous compounds such as heparin.^125^ The structural characteristics of heparin provide multiple potential binding sites for proteins containing heparin-binding domains, facilitating primarily electrostatic interactions that extend beyond coagulation pathways.^126^

UFH and, to some extent, LMWH associate with positively charged proteins at the negatively charged glycocalyx, competitively displacing molecules such as lipoprotein lipase, TF pathway inhibitor, and platelet factor 4.^127^ In addition, heparins display diverse anti-inflammatory effects by binding to IL-6, the IL-6/IL-6 receptor complex, interferon-gamma, and other inflammatory proteins, including chemokines, cytokines, and complement factors.^128^ The overall effect of heparin administration is thought to include anti-inflammatory actions; however, these properties have not been shown in clinical trials to confer additional therapeutic benefit in the management of thromboinflammatory diseases.

**Figure 2:**
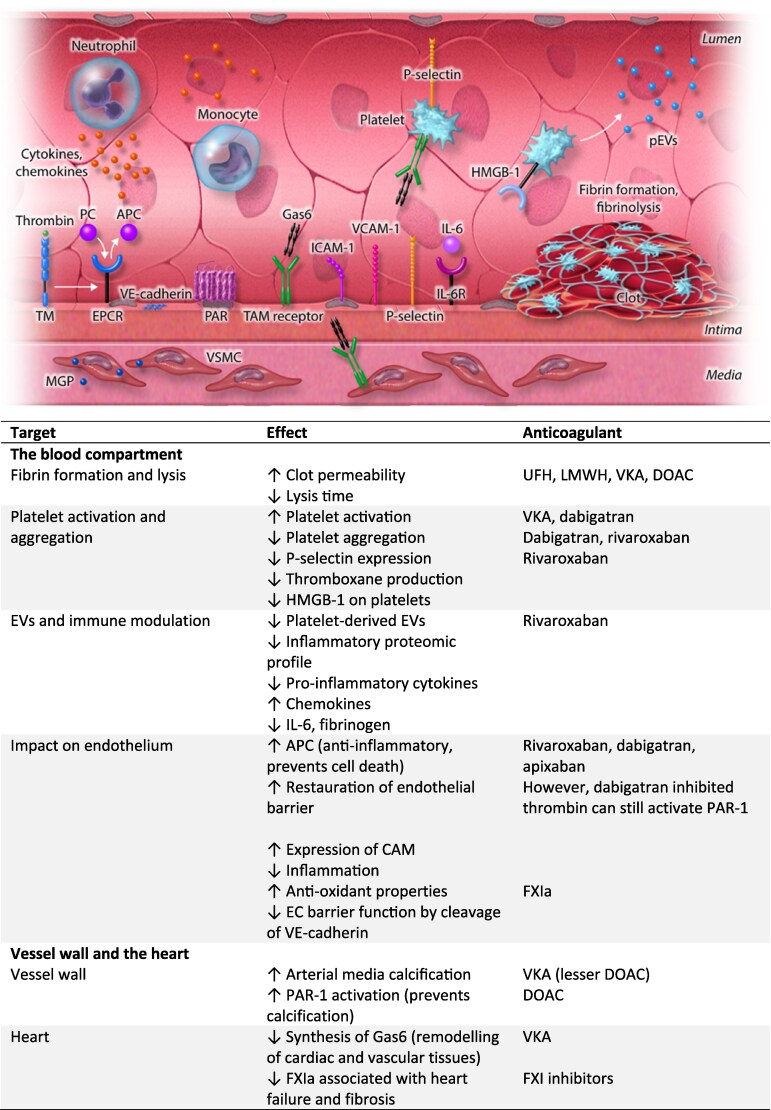
Unintended effects of anticoagulants. APC, activated protein C; CAM, cellular adhesion molecule; DOAC, direct oral anticoagulant; EC, endothelial cell; EPCR, endothelial protein C receptor; EVs, extracellular vesicles; Gas6, growth arrest-specific 6; HMGB-1, high mobility group box 1; ICAM, intercellular adhesion molecule; IL, interleukin; LMWH, low-molecular-weight heparin; MGP, matrix Gla protein; PAR, protease-activated receptor; PC, protein C; TAM, Tyro3, Axl and Mer; TM, thrombomodulin; UFH, unfractionated heparin; VCAM, vascular cell adhesion molecule; VE-cadherin, vascular endothelial cadherin; VKA, vitamin K antagonist; VSMC, vascular smooth muscle cell.

An essential endogenous anticoagulant pathway is the protein C system, which is predominantly activated at the endothelial surface through the binding of thrombin to thrombomodulin and endothelial cell protein C receptor (EPCR) (*Figure 2*). Activated protein C (APC) exerts anti-inflammatory, cytoprotective, and endothelial barrier-stabilizing effects, thereby preventing endothelial cell injury and death.^129,130^ As noted earlier, initiating VKAs can induce a transient hypercoagulable state due to a relative depletion of APC, particularly in individuals with a congenital or acquired protein C deficiency. Whether the generation of APC, and consequently its endothelium protective actions, is differentially regulated by inhibition of thrombin generation via FXa inhibitors or DTIs, when compared with VKAs, is unknown. It is conceivable that residual thrombin activity may continue to support protein C activation, or that alternative APC/EPCR/PAR signalling mechanisms contribute to maintaining endothelial integrity.^131^

VKA-induced uncarboxylated proteases FXa and thrombin, still retain catalytic activity against PARs, although with less efficiency given the impaired phospholipid localization. Consistent with this, recombinant Gla-domainless FXa and thrombin have been shown to activate PARs, and similarly, both APC and Gla-domainless APC can activate PAR-1 in human umbilical vein endothelial cells (HUVECs), provided that the receptor is not shielded by lipid raft localization. Whether such protease-receptor interactions persist and remain physiologically relevant *in vivo* during VKA therapy remains to be determined.^132^

The effects of DOACs on endothelial cells have attracted significant research interest.^133^ In *in vitro* studies with HUVECs, oxysterol reduced endothelial cell integrity and promoted inflammation. Treatment with rivaroxaban or dabigatran, however, appeared to restore endothelial barrier function by upregulating endothelial adhesion molecules such as vascular endothelial (VE)-cadherin, while simultaneously attenuating inflammation through downregulation of intercellular adhesion molecule-1 expression.^134^  ^,135^ Similarly, in an *in vitro* model of uraemia, apixaban prevented endothelial dysfunction and exhibited anti-inflammatory and antioxidant properties.^136^ An *in vitro* study of human brain endothelial cells showed that thrombin-mediated cleavage of PAR-1 altered the expression of tight junction proteins towards a negative effect on endothelial cell integrity. PAR-1 cleavage by thrombin was diminished by pretreatment with dabigatran and rivaroxaban, but not by warfarin or heparin.^137^

As discussed previously, FXa and thrombin interact with PAR-1 and PAR-2 at the vascular endothelium, with their effects shaped by local enzyme concentration and the functional reserve of natural anticoagulant receptors thrombomodulin and EPCR (see previous discussion on mechanisms in Ten Cate *et al*.^138^). Recent data show that rivaroxaban attenuates NLRP3 inflammasome activation via PARs, mitogen-activated protein kinase, and nuclear factor kappa B pathways in a diabetic mouse model and in isolated arterial endothelial cells.^139^ Additional anti-atherogenic effects may include reversal of FXa-mediated suppression of macrophage autophagy, as demonstrated in an atherosclerosis mouse model.^140^

Several *in vivo* studies have explored the effects of DOACs on endothelial function. One randomized controlled trial involving patients with type 2 diabetes and high cardiovascular risk reported that low dose rivaroxaban improved endothelial function, as assessed by enhanced post-ischaemic forearm blood flow, a numerical increase in skin blood flow, and reduced soluble P-selectin compared with aspirin alone. Both groups showed a significant increase in platelet-derived microparticles (PMPs), but PMPs from rivaroxaban-treated patients stimulated HUVEC proliferation more than aspirin. However, no differential effects on biomarkers of inflammation, endothelial cell activation, or vascular stiffness measurements, were noted.^141^ Another study in individuals with chronic heart failure and AF observed improved endothelial function after switching from VKAs to DOACs, as assessed by flow-mediated dilation.^142^ Together, these *in vitro* and *in vivo* findings indicate that DOACs may contribute to endothelial cell integrity by reducing endothelial and inflammatory activation.

FXI may exert distinct effects on the endothelium. Endothelial barrier integrity is regulated by the endothelial cell-specific proteins ROBO-4 and VE-cadherin. FXIa enhances cleavage of VE-cadherin by the metalloproteinase ADAM10, thereby enhancing loss in endothelial cell barrier function. Theoretically, inhibiting FXIa may be beneficial in supporting VE-cadherin function under stress conditions like sepsis.^143^

## 5. Impact of anticoagulants in the vessel wall and the heart

### 5.1 Venous thrombosis and post-thrombotic syndrome

Following inflammatory endothelial damage associated with deep vein thrombosis (DVT), re-endothelialization occurs as part of the thrombus resolution process. In a rat model of DVT, treatment with LMWH was shown to significantly accelerate re-endothelialization during the early phase of thrombus resolution.^144^ Supporting these findings, another study investigating DVT in rats reported that LMWH administration resulted in a two-fold reduction in vein wall stiffness compared with untreated controls.^145^ In earlier work, both LMWH administration and P-selectin inhibition were associated with decreased vein wall stiffness.^146^ However, it remains unclear whether these effects are mechanistically related. Notably, LMWHs have been shown to stabilize the endothelial glycocalyx and inhibit its shedding during inflammation.^147^ By preserving endothelial integrity, LMWHs reduce leukocyte adhesion and vascular permeability, thereby contributing to vascular protection and improved thrombus resolution.

As noted earlier, DOACs may beneficially modify both the structural and functional characteristics of fibrin clots, thereby promoting thrombus resolution. These anticoagulant effects may contribute to the prevention of post-thrombotic syndrome (PTS), a complication of DVT in which residual vein obstruction (RVO) is thought to play a pathophysiological role.^73^ PTS develops as a consequence of persistent venous obstruction, venous hypertension, inflammation, and endothelial activation.^148^ In patients with PTS, fibrin clot permeability is reduced and clot lysis time is prolonged compared with patients with DVT who do not develop PTS.^149^ These findings suggest that the modulation of fibrin clot properties and the shortening of clot lysis time by DOACs may confer clinical benefits by reducing the risk of PTS in patients with DVT.

Interestingly, despite similar rates of RVO, the incidence of PTS was lower in patients treated with DOACs compared with those receiving VKAs in two cohort studies.^150,151^ This observation indicates that the efficacy of DOACs in preventing PTS may involve additional mechanisms beyond the reduction of RVO. A plausible explanation lies in the pathophysiology of PTS itself, which is driven by persistent inflammation leading to endothelial activation and dysfunction.^148^ Given the anti-inflammatory properties of DOACs, these agents may indirectly preserve endothelial function and thereby contribute to their beneficial impact on PTS prevention.

### 5.2 Atherosclerosis and atherothrombosis

Although the anti-inflammatory properties of LMWHs make them an attractive therapeutic option in arterial vascular disease, their requirement for parenteral administration limits long-term use, except in specific circumstances such as pregnancy.

Long-term use of VKAs and DOACs reduces the vascular burden associated with excessive thrombin generation. However, VKAs may promote arterial medial calcification by inhibiting the γ-carboxylation of vascular smooth muscle cell-derived proteins. In contrast, more potent suppression of PAR-1 activation through thrombin inhibition in endothelial cells has been proposed as a mechanism by which DOACs may prevent vascular calcification. By selectively inhibiting FXa and thrombin, DOACs attenuate PAR-1-mediated vascular smooth muscle cell migration and proliferation.^152^

Several observational studies have linked VKA use with an increased risk of vascular calcification and greater coronary plaque burden.^153–155^ This is driven by VKA-induced reductions in vitamin K, which serves as a cofactor for the transformation of vitamin K-dependent proteins. For MGP in particular, impaired γ-carboxylation diminishes its ability to inhibit calcium deposition and crystallization within the vessel wall.^29^

One study reported a higher incidence of vascular calcification in patients on warfarin or rivaroxaban compared with those without anticoagulation, while no such increase was seen on dabigatran.^156^ Moreover, two randomized trials associated VKA use with coronary plaque progression over 52 weeks, compared with apixaban and rivaroxaban.^157,158^ In contrast, another randomized trial found no significant difference in coronary calcification progression between VKAs and rivaroxaban after 12 and 24 months.^159^ Collectively, these findings suggest that DOACs may confer an advantage over VKAs in mitigating vascular calcification. However, it remains uncertain whether this effect is consistent across different vascular territories or whether specific DOACs, such as dabigatran, offer superior protection compared with FXa inhibitors.

As discussed in previous articles, also in this journal, DOACs exert notable effects on the vasculature, particularly by inhibiting the progression or promoting the regression of atherosclerosis in experimental mouse models.^138,160,161^ Furthermore, interference with the protease-PAR signalling properties of FXa and thrombin may profoundly influence smooth muscle cell function, thereby modulating the balance between physiological vascular repair and pathological remodelling.^162^ Owing to their small molecular size and substantial volume of distribution, particularly for dabigatran, DOACs may penetrate tissues efficiently and exert significant extravascular effects.

The concept of tissue-penetrating properties is exemplified in tumour models, where tumour-associated macrophages have been shown to produce FXa, thereby activating local FXa-PAR-2 signalling pathways that promote tumour growth and progression.^163^ Inhibition of this pathway may, in turn, reprogram tumour-associated macrophages, enhancing tumour antigen presentation and facilitating immune-mediated tumour cell destruction in certain experimental models.^164,165^

These examples illustrate the broader principle that tissue function and repair can be modulated by protease inhibitors such as DOACs. Additional studies have demonstrated intracellular effects beyond anticoagulation, including dabigatran in experimental models of asthma and Alzheimer’s disease, rivaroxaban influencing mitochondrial proteins in models of aortic aneurysm, and dose-dependent antioxidant effects of both rivaroxaban and edoxaban in mitochondria derived from renal cells.^166–170^ Recent prospective data aligns with these mechanistic insights, showing that patients with AF and chronic kidney disease experienced fewer adverse kidney outcomes when treated with rivaroxaban compared with VKAs.^171^ It remains uncertain whether this difference reflects VKA-associated vascular injury and calcification, anti-inflammatory effects of rivaroxaban, or an interplay of both mechanisms.

Clinically, evidence for vascular pleiotropic effects of DOACs are still limited. A retrospective analysis suggested reduced restenosis rates following femoral-popliteal artery stenting in patients receiving DPI compared with those on dual antiplatelet therapy. Another small cohort study, using low dose rivaroxaban with clopidogrel instead of aspirin, also reported improved patency and reduced restenosis, without impact on clinical outcomes like amputation rates.^172,173^

Therapeutic doses of rivaroxaban or dabigatran did not significantly affect intima media thickness or biomarkers of atherosclerosis and endothelial dysfunction compared with warfarin after 12 or 24 months in patients with AF.^174^ Switching from prolonged VKA therapy to rivaroxaban led to a reduction in brachial-ankle pulse wave velocity after three months, compared with continued VKA use.^175^ However, flow mediated dilation of the brachial artery in patients on long-term VKA or apixaban therapy showed no significant differences between groups.^176^

Taken together, these limited data from small human studies with relatively short follow-up periods do not provide clear evidence for vascular protective effects of DOACs at either the macrovascular or microvascular level. Nonetheless, such effects may emerge after prolonged exposure or may be restricted to individuals with specific characteristics, such as enhanced inflammation or underlying dyslipidaemia, comparable to atherogenic mouse models used in preclinical DOAC research.

Similarly to DOACs, targeting FXI with either a monoclonal antibody (14E11) or an antisense oligonucleotide reduced atherosclerosis burden in mouse studies. In addition, 14E11 prevented disruption of endothelial cell VE-cadherin in the aortic sinus, suggesting a FXI-specific effect, although a contribution of reduced thrombin generation cannot be excluded.^177^ One specific feature may be that, in mice, most FXI is associated with the endothelial glycocalyx, implying a greater local functional effect than in larger species like baboons and humans.^178^ Nevertheless, given that FXI may be involved in diverse inflammatory mechanisms at or beneath the endothelial cell surface in humans, interfering in the function of this protein may have wider implications beyond inhibition of the downstream intrinsic cascade.^179^

### 5.3 The heart

VKAs remain the preferred OAC for high risk thrombosis patients, which may be due to a more potent anticoagulant effect.^180^ Another specific VKA effect involves the reduced synthesis of vitamin K-dependent proteins in tissues, including Gas6.^181^ VKAs inhibit the γ-carboxylation of Gas6, a ligand for the TAM family of tyrosine kinase receptors.^182,183^

The Gas6/Axl pathway may be implicated in remodelling processes in cardiac and vascular tissues.^183^ In Gas6 knockout mice, chronic pressure overload induced by aortic banding resulted in reduced cardiac hypertrophy, fibrosis, and contractile dysfunction compared with wild-type controls, whereas Gas6 overexpression induced an adverse phenotype. In hypertrophic murine hearts, Gas6 expression was upregulated in comparison with control hearts. Similarly, Gas6 expression was upregulated in the hearts of patients with dilated cardiomyopathy compared with donor hearts.^184^ The DRAGON-HF trial linked elevated plasma Gas6 concentrations in patients with acute heart failure to a higher all-cause and cardiovascular mortality.^185^

Taken together, a better understanding of VKA’s pleiotropic effects is important to inform future therapeutic strategies targeting specific intermediates in the Gas6 pathway.

In an *in vitro* study with human cardiac fibroblasts, incubation with FXa led to overexpression of pro-inflammatory genes via PAR-1, suggesting DOACs may exert cardioprotective effects by inhibiting FXa/thrombin dependent PAR-1 activation.^186^ Ischaemia-reperfusion injury after myocardial infarction is characterized by an inflammation response and apoptosis, involving a complex and critical interaction between inflammation and coagulation. In animal models, natural anticoagulants have demonstrated cardioprotective functions.^187^ Compared with untreated controls, mice treated with rivaroxaban and dabigatran showed reduced infarct sizes after induction of ischaemia-reperfusion injury, but only rivaroxaban showed an anti-inflammatory gene expression profile.^188^

Experiments have linked FXI to cardiac function, which is relevant given the ongoing developments of FXI inhibitors. In a mouse model of diastolic heart failure, FXI overexpression in the liver improved diastolic function, whereas FXI knockout mice exhibited increased severity. This effect of FXI may be related to bone morphogenetic protein 7 (BMP7), which is present in significant concentrations within cardiac tissue. FXIa cleaves BMP7 in the extracellular matrix in the heart, subsequently activating the BMP7-SMAD1/5 signalling pathway. As a result, genes involved in inflammation and fibrosis are inhibited.^189^ In a large community-based cohort study, lower plasma FXI levels were associated with a higher incidence of heart failure, particularly among participants aged ≥75 years.^190^ This association was validated in the Cardiovascular Health Study.^190^ However, it remains unclear whether this age-related risk is confounded by other risk factors for heart failure or if the effect of decreased plasma FXI levels depends on duration of exposure.

One risk factor both for atherosclerosis and heart failure is hypertension. Translational studies showed that platelet-localized FXI plays a role in accelerated thrombin generation, associated with and possibly causally involved in uncontrolled hypertension.^191^ Although a link between plasma FXI(a) levels and hypertension has not yet been reported from clinical trials of FXI inhibitors, recent research is exploring FXI inhibition as a possible antihypertensive strategy.^192^

## 6. Concluding remarks

Over the years, anticoagulant therapy has evolved significantly, primarily focusing on preventing and treating thrombosis by inhibiting thrombin generation and subsequent fibrin formation. Beyond their established anticoagulant effects and associated bleeding risks, accumulating evidence highlights important pleiotropic effects both outside and inside the cardiovascular system, involving interactions with platelets, leukocytes, cytokines, chemokines, EVs, endothelial cells, vascular smooth muscle cells, endothelial receptors, and signalling pathways.

Overall, the dynamic landscape of anticoagulation therapy reveals that, in addition to their intended anticoagulant targets, pleiotropic effects contribute to therapeutic outcomes. Long-term data on pleiotropic effects of DOACs and FXI inhibitors are still limited, highlighting the need for further research to determine their clinical implications and whether certain patient populations may benefit from specific pleiotropic effects of anticoagulants.
